# Aerobic versus resistance exercises on systemic inflammation and sleep parameters in obese subjects with chronic insomnia syndrome

**DOI:** 10.4314/ahs.v21i3.30

**Published:** 2021-09

**Authors:** Osama H Al-Jiffri, Shehab M Abd El-Kader

**Affiliations:** 1 Department of Medical Laboratory Technology, Faculty of Applied Medical Sciences, King Abdulaziz University, Jeddah, Saudi Arabia; 2 Department of Physical Therapy, Faculty of Applied Medical Sciences, King Abdulaziz University, Jeddah, Saudi Arabia

**Keywords:** Aerobic exercise, chronic primary insomnia, inflammatory cytokines, resistance exercise, sleep quality

## Abstract

**Background:**

Chronic primary insomnia is a prevalent sleep disorder that is associated with adverse effects on health outcomes. Exercise is often considered a non-pharmacological approach that could have beneficial effects on sleep.

**Objective:**

The aim of the study was to compare the impact of aerobic and resistance exercise training on quality of sleep and inflammatory markers among subjects with chronic primary insomnia.

**Material and methods:**

Sixty previously sedentary subjects with chronic primary insomnia subjects enrolled in this study, their age ranged from 31–52 years. All participants were randomly assigned to aerobic exercise intervention group (group A, n=35) or resistance exercise intervention group (group B, n=35). Polysomnographic recordings for sleep quality assessment, IL-6, IL-10 and TNF-α were measured before and at the end of the study after six months.

**Results:**

There was a significant increase in the total sleep duration, sleep efficiency, sleep onset latency and IL-10 in group(A) and group (B) in addition to significant reduction in awake time after sleep onset, REM latency, IL-6 and TNF-α after 6 months of aerbic and resistance exercise training. However, there were significant differences between both groups at the end of the study.

**Conclusion:**

Aerobic exercise training is more appropriately than resistance exercise training in modulation of inflammatory and sleep quality among subjects with chronic primary insomnia.

## Introduction

Insomnia is prevalent sleep disorder plaguing an estimated 15% of the population 1,2. However, insomnia is associated with deleterious effects on health, such as increased incidence of all-cause mortality, coronary artery disease, type 2 diabetes mellitus and hypertension^3^,^4^. Moreover, impaired sleep is also linked to changes in metabolism, increased caloric intake, and obesity 5,6.

Sleep disruption show subsequent increases in blood pressure and elevation of inflammatory cytokines, including those implicated in atherogenesis such as C-reactive protein (CRP), tumor necrosis factor-α and interleukin 7–9. Low grade inflammation is, in part, responsible for the increased rate of coronary heart disease found in this population 10,11. Likewise, inflammatory processes are a hallmark etiological factor in cancer development and progression and are expressed not only at tumor sites but also peripherally 12,13. There is now increasing evidence from clinical studies that peripheral levels of pro-inflammatory biomarkers, (e.g., CRP) are predictive of cancer risk and survival 14.

Several studies have tested using exercise as a nondrug treatment for insomnia, where results of these studies suggested that long-term (three months or longer) exercise could contribute to better sleep quality or eased insomnia symptoms among insomniac populations^15^–^17^. Aerobic exercise shown to improve both sleep quantity and quality 18,19. Progressive resistance exercise is an alternative modality that has also been shown to improve sleep quality 20,21. Like aerobic exercise, resistance exercise has been shown to improve comorbidities commonly associated with poor sleep, such as depression 22 and cardiovascular disease 23, and may be suitable in those for whom aerobic exercise is not feasible or desirable. To our knowledge, the differences between the effects of resistance and aerobic exercise on sleep outcomes and inflammatory markers among subjects with insomnia has not been published. Thus, the purpose of this investigation was to compare the impact of aerobic and resistance exercise training on quality of sleep and inflammatory markers among subjects with Chronic Primary Insomnia.

## Patients and methods

### Subjects

**Figure (1):**
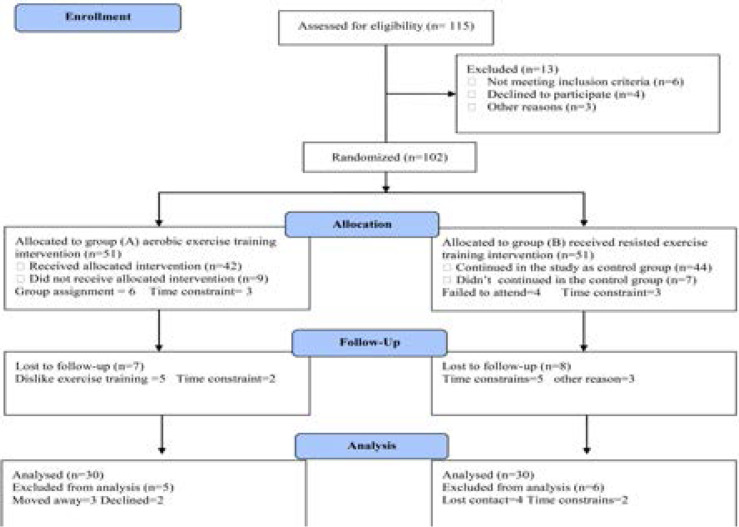
Subjects screening and recruitment CONSORT diagram

Sixty previously sedentary subjects having chronic primary insomnia for longer than six months, their age ranged from 35–56 years and participated in this study. Exclusion criteria included history of use of psychotherapeutic drugs, shiftwork, exercise training for more than one day /week, smoking, alcohol abuse, major psychiatric disorders and caffeine intake more than 300 mg/day. All subjects were cleared for participation by their personal physician, reported willingness to be randomly assigned to treatment conditions, and agreed not to participate in exercise outside the study. No attempts were made to control dietary intake. Subjects were randomized to either an aerobic exercise intervention group (group A) or resistance exercise intervention group (group B). Both groupsparticipated in the exercise intervention conducted 3 times per week for 6 months. Exercise sessions were supervised and monitored by trained exercise specialists. The CONSORT diagram display the essential details of randomization (figure 1). Informed consent was obtained from all participants. This study was approved by the Scientific Research Ethical Committee, Faculty of Applied Medical Sciences at King University.

## Methods

### Measurements

The following measurements were taken before the study and after 6 months at the end of the study.

A. Sleep measures: All participants underwent polysomnographic (PSG) recording before and after the exercise training. For the pre-intervention assessment, PSG recording was performed over 2 nights. The room used for the recordings had a large comfortable bed, acoustic isolation, and controlled temperature and light. Recordings were conducted by a trained sleep technician using a digital system (Philips-Respironics, USA) 24.

B. Inflammatory cytokines: Blood samples were drained from the antecubital vein after a 12-hour fasting, the blood samples were centrifuged at + 4 °C (1000 = g for 10 min). “Immulite 2000” immunassay analyzer (Siemens Healthcare Diagnostics, Deerfield, USA) analyzed Interleukin-6 (IL-6) and Interleukin-10 (IL-10) levels. However, tumor necrosis factor-alpha (TNF-α) was measured by ELISA kits (ELX 50) in addition to ELISA microplate reader (ELX 88; BioTek Instruments, USA).

### Procedures

Following the previous evaluation, all patients will be divided randomly into the following groups:

A. Aerobic exercise training program: Patients in group (A) were submitted to a 40 min aerobic session on a treadmill (the initial, 5-minute warm-up phase performed on the treadmill at a low load, each training session lasted 30 minutes and ended with 5-minute recovery and relaxation phase) either walking or running, based on heart rate, until the target heart rate was reached, according to American College of Sport Medicine guidelines. The program began with 10 min of stretching and was conducted using the maximal heart rate index (HRmax) estimated by 220-age. First 3 months = 60–70% of HRmax, second 3 months = 70–80% of HRmax 25

B. Resistance exercise training: Patients of group (B) were submitted to a 40 min session of resistance training. The program began with 10 min of stretching and was conducted with exercises done on nine resistance machines. The resistance machines used were: chest press, bicep curl, triceps extension, lower back, abdominals, leg press, leg curl and leg extension. Subjects performed three sets of 8–12 repetitions, with 60 s of rest between each set. Resistance was increased by five pounds after the subject was able to complete three sets of eight repetitions on three consecutive days. Subjects were trained using between 60 and 80% of their one maximal repetition weight (1-RM) 26

### Statistical analysis

The mean values of the investigated parameters obtained before and after three months in both groups were compared using paired “t” test. Independent “t” test was used for the comparison between the two groups (P<0.05).

## Results

The two groups were considered homogeneous regarding the demographic variables (table 1). The mean age of group, (A) was 46.12 ± 3.65 years, and the mean age of group (B) was 44.87 ± 4.32 years. There was no significant differences in age, gender, body mass index (BMI), body fat, systolic blood pressure, diastolic blood pressure, hemoglobin and maximal heart rate (HRmax) between both groups.

**Table (1):** Baseline characteristics of study participants

Characteristic	Group (A)	Group (B)	Significance
**Age** (years)	43.64 ± 3.97	41.51 ± 4.26	P>0.05
**Gender** (male/female)	18/12	20/10	P>0.05
**BMI** (kg/m^2^)	34.19 ± 3.41	33.28 ± 3.92	P>0.05
**Waist hip ratio**	0.89 ± 0.27	0.88 ± 0.25	P>0.05
**SBP** (mmHg)	137.83 ± 8.14	135.92 ± 7.69	P>0.05
**DBP** (mmHg)	86.31 ± 5.16	84.72 ± 4.91	P>0.05
**Hb** (gm/dl)	11.74 ± 1.68	12.25± 1.42	P>0.05
**HR_max_** (beat/min)	162.85 ± 13.12	164.76 ± 14.35	P>0.05

BMI: Body mass index; SBP: Systolic blood pressure; DBP: Diastolic blood pressure; Hb: Hemoglobin; HR_max_: Maximum heart rate.

There was a significant reduction in BMI, CD3, CD4 and CD8 awake time after sleep onset, REM latency, IL-6 and TNF-α in addition to significant increase in the total sleep duration, sleep efficiency, sleep onset latency and IL-10 after 6 months of in group(A) as a result of weight loss program (table 2); while the results of the control group (group B) were not significant (table 3). Also, there were significant differences between both groups at the end of the study(table 4).

**Table (2):** Mean value and significance of sleep parameters and inflammatory markers of group (A) before and at the end of the study

	Mean + SD		t-value	Significance
	Pre	Post		
**BMI** (kg/m^2^)	34.19 ± 3.41	28.26 ± 3.12*	7.95	P<0.05
**Total sleep** **duration** (min)	320.67 ± 25.92	354.23 ± 28.51*	11.46	P <0.05
**Sleep efficiency** (%)	67.85 ± 6.34	83.32 ± 7.19*	10.13	P <0.05
**Sleep onset latency** (min)	12.37 ± 2.68	16.11 ± 2.75*	7.26	P <0.05
**Awake time after sleep** **onset** (min)	78.91 ± 7.54	63.25 ± 6.82*	9.67	P <0.05
**REM sleep latency** (min)	90.13 ± 8.63	71.27 ± 6.94*	10.21	P <0.05
**TNF-α** (pg/mL)	6.17 ± 1.82	3.75 ± 1.41*	6.83	P <0.05
**IL-6** (pg/mL)	2.76 ± 0.84	1.69 ± 0.73*	5.72	P <0.05
**IL-10** (pg/ml)	5.91 ± 1.35	8.22 ± 1.64*	6.43	P <0.05

BMI: Body mass index; REM: rapid eye movements; TNF-α: tumor necrosis factor – alpha; IL-6: Interleukin-6; IL-10: Interleukin-10

* indicates a significant difference between the two groups, P < 0.05.

**Table (3):** Mean value and significance of sleep parameters and inflammatory markers of group (B) before and at the end of the study

	Mean + SD		t-value	Significance
	Pre	Post		
**BMI** (kg/m^2^)	33.28 ± 3.72	33.75 ± 3.78	0.481	P>0.05
**Total sleep** **duration** (min)	323.74 ± 27.19	319.24 ± 26.97	1.82	P>0.05
**Sleep efficiency** (%)	69.15 ± 5.84	68.21 ± 5.76	1.17	P>0.05
**Sleep onset latency** (min)	12.71 ± 2.53	12.15 ± 2.68	0.614	P>0.05
**Awake time after sleep** **onset** (min)	76.54 ± 6.91	78.13 ± 7.11	1.15	P>0.05
**REM sleep latency** (min)	88.75 ± 8.42	89.66 ± 8.54	0.871	P>0.05
**TNF-α** (pg/mL)	5.98 ± 1.75	6.11 ± 1.81	0.493	P>0.05
**IL-6** (pg/mL)	2.65 ± 0.71	2.94 ± 0.78	0.476	P>0.05
**IL-10** (pg/ml)	6.13 ± 1.47	5.86 ± 1.43	0.392	P>0.05

BMI: Body mass index; REM: rapid eye movements; TNF-α: tumor necrosis factor – alpha; IL-6: Interleukin-6; IL-10: Interleukin-10.

**Table (4):** Mean value and significance of sleep parameters inflammatory markers in group (A) and group (B) at the end of the study

	Mean + SD		t-value	Significance
	Group (A)	Group (B)		
**BMI** (kg/m^2^)	28.26 ± 3.12*	33.75 ± 3.78	6.19	P<0.05
**Total sleep** **duration** (min)	354.23 ± 28.51*	319.24 ± 26.97	9.24	P <0.05
**Sleep efficiency** (%)	83.32 ± 7.19*	68.21 ± 5.76	8.23	P <0.05
**Sleep onset** **latency** (min)	16.11 ± 2.75*	12.15 ± 2.68	6.35	P <0.05
**Awake time after sleep** **onset** (min)	63.25 ± 6.82*	78.13 ± 7.11	7.56	P <0.05
**REM sleep** **latency** (min)	71.27 ± 6.94*	89.66 ± 8.54	8.48	P <0.05
**TNF-α** (pg/mL)	3.75 ± 1.41*	6.11 ± 1.81	5.27	P<0.05
**IL-6** (pg/mL)	1.69 ± 0.73*	2.94 ± 0.78	4.75	P<0.05
**IL-10** (pg/ml)	8.22 ± 1.64*	5.86 ± 1.43	5.18	P<0.05

BMI: Body mass index; REM: rapid eye movements; TNF-α: tumor necrosis factor – alpha; IL-6: Interleukin-6; IL-10: Interleukin-10

* indicates a significant difference between the two groups, P < 0.05.

## Discussion

Insomnia is one of the most common sleep disorders and is a risk factor for future cardiac events, including acute myocardial infarction and coronary heart disease, even among individuals free of cardiovascular disease^27^. However, exercise promoted increased sleep efficiency and duration in populations suffering from chronic sleep complaints 28,29. Concerning sleep quality parameter, the results of the present study revealed that there was a significant increase in the total sleep duration, sleep efficiency and sleep onset latency in group(A) and group (B) in addition to significant reduction in awake time after sleep onset and REM latency after 6 months of aerobic and resistance exercise training. However, there were significant differences between both groups at the end of the study, these results are in line with many previous studies as Reid and colleagues had Seventeen sedentary elderly subjects with insomnia who had 16 weeks of aerobic physical activity. The clearly stated that physical activity improved sleep quality on the global Pittsburgh Sleep Quality Index (PSQI) score, sleep latency, sleep duration, daytime dysfunction and sleep efficiency 30. Where, Lira et al. conducted a study on fourteen male sedentary volunteers performed moderate training for 60 minutes/day, 3 days/week for 24 weeks at a work rate equivalent to the ventilatory aerobic threshold. They proved that sleep parameters, awake time and REM sleep latency were decreased after 6 months exercise training in relation baseline values 31.

Yang and colleagues completed a systematic review with meta-analysis of six randomized trials and provided data on 305 participants (241 female). Each of the studies examined an exercise training program that consisted of either moderate intensity aerobic exercise or high intensity resistance exercise. The duration of most of the training programs was between 10 and 16 weeks. All of the studies used the self-reported Pittsburgh Sleep Quality Index to assess sleep quality. Compared to the control group, the exercise group had significantly reduced sleep latency and medication use^32^. While, Chen and coworkers enrolled twenty-seven participants in 12 weeks of exercise training, they proved that overall sleep quality, subjective sleep quality, sleep latency, sleep duration, sleep efficiency, and daytime dysfunction significantly improved after 12 weeks of intervention^33^. In addition, Santos et al. had twenty-two male, sedentary volunteers performed moderate training for 60 min/day, 3 days/week for 24 week at a work rate equivalent to their ventilatory aerobic threshold, their findings suggest that aerobic exercise training increased aerobic capacity parameters, decreased REM latency and decreased time awake^34^. Moreover, Passos and colleagues concluded that a 4-month intervention of moderate aerobic exercise delivered to twenty-one sedentary participants with chronic primary insomnia had polysomnographic data significantly improvements following exercise training, where total sleep time, sleep efficiency and rapid eye movements significantly increased. In addition, sleep onset latency and wake time after sleep onset significantly decreased following exercise training 35.

Tan and co-workers enrolled 45 obese Finnish men with chronic insomnia symptoms in a six-month aerobic exercise program and resulted showed that sleep efficiency and quality improved significantly 36. Similarly, Ferris et al. in a study with a resistance exercise protocol quite similar to ours but conducted on only eight elderly subjects aged 78 years on average, applied six exercises for the upper and lower limbs with 10–12 repetitions at 50% 1 RM over a period of six months and reported that resistance exercise imprved sleep parameters^37^. In the other hand, two previous studies in older adults reported a small-to-moderate positive effect on sleep duration 38,39. The remaining study in younger adults with insomnia reported a large but non-significant negative effect on sleep duration following moderate-intensity resistance training 40.

Regarding, the mechanism underlying the effect of exercise on sleep, although the mechanisms by which training can improve sleep quality are not well understood. It has been proposed that exercise training improves sleep quality through increasing energy consumption, endorphin secretion, or body temperature in a manner that facilitates sleep for recuperation of the body 41–43. In addition, some other mechanisms, such as an increasing in energy consumption, endorphin secretion, body temperature, are also beneficial to improve sleep quality 44. Moreover, moderate training may reduce resting plasma concentrations of pro-inflammatory cytokines and increase anti-inflammatory cytokines, consequently improving the quality of sleep 45.

Our results demonstrate that both aerobic and resistance exercise training causes a decrease in TNF-α, IL-6 and CRP levels, in addition to increase in IL-10 level which suggests that exercise training can reduce inflammation with more significant changes following aerobic exercise training. Several studies have shown that moderate physical exercise promotes the modulation of inflammation^46^–^48^. Several large cohort studies have found a relationship between self-reported physical activity levels and systemic markers of inflammation: higher levels of physical activity are coupled to lower levels of circulating inflammatory markers in elderly individuals^49^–^51^. Regarding the aerobic exercise training, our results agreed with Nicklas et al. showed that regular aerobic exercise training was efficient in lowering IL-6 levels even without weight loss 52. Also, Santos and colleagues had twenty-two male, sedentary, healthy, elderly volunteers performed moderate aerobic exercise training for 60 min/day, 3 days/week for 24 week and concluded that 6 months of aerobic exercise training can improve sleep in the elderly via anti-inflammatory effect of aerobic training which modifies cytokine profiles (reduced IL-6 and TNF-α and increased IL-10) 53. In addition, Salamat and colleagues reported significant difference in IL-6 between endurance and resistance groups that following 8 weeks of training in overweight men and concluded that endurance and concurrent exercise training in part has a positive effect on pre-inflammatory cytokines 54.

In the other hand, Kohut et al. reported that 10-months of aerobic, but not resistance exercise, significantly reduces serum inflammatory mediators in older adults 55. In addition, Bote et al. demonstrated that 8-months (2 sessions/week, 60-min/session) of aquatic-based exercise training tempered neutrophil activation (chemotaxis) and decreased systemic levels of IL-8 and noradrenalin compared to controls 56. However, our results regarding resistance exercise training agreed with White et al. found alterations in the biomarkers of inflammation after 8 weeks of resistance training in individuals with multiple sclerosis 57. Where, Prestes et al. performed a resistance training for 16 weeks in elderly sedentary and found reductions in the levels of IL-6 after training 58. Moreover, our results confirmed that aerobic exercise training is more appropriate to modify the inflammatory markers among elderly and these agreed with Ploeger et al. reported that moderate aerobic exercise training has been recommended as an anti-inflammatory therapy^59^.

The three possible mechanisms of exercise anti-inflammatory effects include reduction in visceral fat mass 60; reduction in the circu¬lating numbers of pro-inflammatory monocytes 61 and an increase in the circulating numbers of regulatory T cells 62. Moreover, Hong and colleagues show that cardiorespiratory fitness is associated with reduced low grade inflammation that may in part be mediated by enhancing the ability of immune cells to suppress inflammatory responses via adrenergic receptors 63.

The current study has important strengths and limitations. The major strength is the supervised nature of the study. However, all exercise sessions were supervised. Moreover, the study was randomized; hence, we can extrapolate adherence to the general population. In the other hand, the major limitations is only obese middle aged subjects were enrolled in the study, so the value of this study only related to obese subjects in this age group, also small sample size in both groups may limit the possibility of generalization of the findings in the present study. Finally, within the limit of this study, aerobic exercise training is recommended for modulation of inflammatory and sleep quality among subjects with Chronic Primary Insomnia. Further researches are needed to explore the impact of weight reduction on quality of life and other biochemical parameters among subjects with Chronic Primary Insomnia.

## Conclusion

Aerobic exercise training is more appropriately than resistance exercise training in modulation of inflammatory and sleep quality among subjects with Chronic Primary Insomnia.
